# Exosomes from Human Gingiva-Derived Mesenchymal Stem Cells Combined with Biodegradable Chitin Conduits Promote Rat Sciatic Nerve Regeneration

**DOI:** 10.1155/2019/2546367

**Published:** 2019-05-02

**Authors:** Feng Rao, Dianying Zhang, Tengjiaozi Fang, Changfeng Lu, Bo Wang, Xiao Ding, Shuai Wei, Yiran Zhang, Wei Pi, Hailin Xu, Yanhua Wang, Baoguo Jiang, Peixun Zhang

**Affiliations:** ^1^Department of Orthopedics and Trauma, Peking University People's Hospital, Beijing, China; ^2^Department of Pediatric Dentistry, Peking University School and Hospital of Stomatology, National Engineering Laboratory for Digital and Material Technology of Stomatology, and Beijing Key Laboratory of Digital Stomatology, Beijing 100081, China; ^3^Institute of Orthopedics, Chinese PLA General Hospital, Beijing Key Lab of Regenerative Medicine in Orthopedics, Beijing, China

## Abstract

At present, repair methods for peripheral nerve injury often fail to get satisfactory result. Although various strategies have been adopted to investigate the microenvironment after peripheral nerve injury, the underlying molecular mechanisms of neurite outgrowth remain unclear. In this study, we evaluate the effects of exosomes from gingival mesenchymal stem cells (GMSCs) combined with biodegradable chitin conduits on peripheral nerve regeneration. GMSCs were isolated from human gingival tissue and characterized by surface antigen analysis and in vitro multipotent differentiation. The cell supernatant was collected to isolate the exosomes. The exosomes were characterized by transmission electron microscopy, Western blot, and size distribution analysis. The effects of exosomes on peripheral nerve regeneration in vitro were evaluated by coculture with Schwann cells and DRGs. The chitin conduit was prepared and combined with the exosomes to repair rat sciatic nerve defect. Histology, electrophysiology, and gait analysis were used to test the effects of exosomes on sciatic nerve function recovery in vivo. We have successfully cultured GMSCs and isolated exosomes. The exosomes from GMSCs could significantly promote Schwann cell proliferation and DRG axon growth. The in vivo studies showed that chitin conduit combined with exosomes from GMSCs could significantly increase the number and diameter of nerve fibers and promote myelin formation. In addition, muscle function, nerve conduction function, and motor function were also obviously recovered. In summary, this study suggests that GMSC-derived exosomes combined with biodegradable chitin conduits are a useful and novel therapeutic intervention in peripheral nerve repair.

## 1. Introduction

Every year, a large number of patients suffer from peripheral nerve injury worldwide, and the lack of effective treatments often leads to disability, which imposes a heavy burden on families and society [1–3]. There are many surgical methods to repair peripheral nerve injuries, and end-to-end anastomosis is usually used for large injuries. Long peripheral nerve defects, from traumatic injuries, often require grafts to bridge the gap. Although autologous nerve transplantation remains the preferred strategy for reconstruction, it is limited by donor tissues, the sacrifice of functional nerves, and the potential formation of neuromas [4–6].

The repair of peripheral nerve injury is a very complicated pathological process, and it is difficult to obtain satisfactory results because of slow nerve regeneration, Wallerian degeneration, tissue adhesion, and muscle and motor endplate atrophy. Wallerian degeneration occurs after peripheral nerve injury, after which macrophages infiltrate the injured nerve and produce large amounts of inflammatory factors including CCL2, TNF-a, IL-1a, and IL-1beta [7, 8].

The development of new therapeutic strategies to accelerate neurite outgrowth is of great importance [9]. Accordingly, mesenchymal stem cells (MSCs) have been used therapeutically for tissue regeneration and to treat autoimmune diseases [10, 11]. Recent evidence suggests that a variety of mechanisms contribute to MSC-based therapies in which cytokines and growth factors can act as paracrine or autocrine mediators to modulate immune responses and tissue regeneration [12, 13].

Exosomes are extracellular vesicles that are secreted by living cells. They are released into the extracellular space through fusion of polyvesicular bodies and cytoplasmic membranes. The diameter of exosomes ranges from 30 to 150 nm, and their size is similar to that of viruses [14]. Further, exosomes are widely involved in intercellular communication and play an important role in tissue repair and regeneration, immune regulation, and organism development [15, 16]. Stem cell-derived exosomes can repair skin defects [17], treat fractures [18], promote cartilage defect repair [19], regulate microglial M1/M2 polarization, enhance brain injury repair [20], and facilitate the outward growth of neural axons after trauma [21].

Vasculogenesis plays an important physiological role in tissue repair. Specifically, it can result in the transport of oxygen, nutrients, and immune cells to the injured site. Moreover, many studies have found that cytogenic exosomes can participate in signaling pathways involved in angiogenesis, affecting the development and maturation of blood vessels [22].

Immunoinflammation plays an important role in coordinating a series of physiological processes involved in tissue repair and regeneration. However, it remains a challenge to control inflammation. Many studies have confirmed that MSC-derived exosomes can mimic the ability of MSCs to regulate immune cells including B cells, T cells, natural killer cells, dendritic cells, and macrophages [23, 24]. Gingival mesenchymal stem cells (GMSCs) are stem cell-specific precursor cells that can be isolated from gingival tissues [25–27] and have the capacity for self-renewal, multidirectional differentiation, and immune regulation. GMSCs present different advantages, including their collection which did not require invasive procedures and allow MSC collection from the patients for autologous transplantation. Moreover, they had different advantages compared to bone marrow-derived MSCs. Indeed, GMSCs are easy to isolate, homogenous, and proliferate faster than bone marrow-derived MSCs, present a stable morphology, and maintain a normal karyotype and MSC characteristics also after long-term cultures [28–30]. However, whether exosomes derived from gingival stem cells can promote peripheral nerve regeneration has not been reported. In this study, we used a biodegradable chitin conduit combined with gingival stem cell-derived exosomes to bridge a 10 mm sciatic nerve defect in rats to address this question.

## 2. Materials and Methods

### 2.1. Culture of Human Gingiva-Derived Mesenchymal Stem Cells

With the approval of the ethical committee of the Peking University Hospital of Stomatology, gingivae were collected from four healthy patients without a history of periodontal disease, such as upon wisdom tooth extraction. Human GMSCs were isolated as described in published protocols [31]. The gingival tissues were treated aseptically, washed several times with PBS, and cultured in a medium containing 2 mg/mL protease (Gibco) at 4°C. After terminating the digestion, the epithelial layer and the lamina propria were separated, and the lamina propria was collected and shredded. Tissues were cut into pieces and digested with collagenase IV at 37°C for 1 hour and centrifuged at 1000 rpm for 10 minutes, and the supernatant was discarded. The cells were suspended in a 10 cm cell culture dish and cultured in a humidified cell culture incubator containing 5% CO_2_ at 37°C for 24 hours in alpha-MEM medium containing 10% FBS and 100 U/mL penicillin/100 *μ*g/mL streptomycin. The original culture medium was discarded, and the floating cells were removed. Fresh medium was added. The medium was then replaced every two days. When cell fusion reached 80%, cells were digested using trypsin for continuous passage. Passages from generations 3–5 were used for experiments.

### 2.2. Flow Cytometric Analysis

Cells (2 × 10^5^) were incubated with a specific monoclonal antibody conjugated with either fluorescein isothiocyanate (FITC) or phycoerythrin (PE) in 200 *μ*L PBS (SH30256.01; HyClone) for 30 min in the dark at 4°C. The cell surface antigens were then analyzed by flow cytometry. Antibodies against CD34 (PE) (ab187284; Abcam), CD45 (PE) (ab134202; Abcam), CD73 (FITC) (ab239246; Abcam), CD90 (FITC) (ab124527; Abcam), and CD146 (FITC) (ab78451; Abcam) were used. Mouse monoclonal IgG1 (ab170190; Abcam) isotype was used as control.

### 2.3. Multipotent Differentiation of GMSCs

#### 2.3.1. Osteogenic Differentiation

GMSCs were plated at 5 × 10^5^ cells/well in 6-well plates in MSC growth medium; upon reaching 60% fusion, medium was replaced with osteogenic induction medium supplemented with dexamethasone, L-glutamine, ascorbic acid, and *β*-glycerophosphate. The culture medium was changed every three days. After 4 weeks, in vitro mineralization was assayed by Alizarin Red S staining.

#### 2.3.2. Adipogenic Differentiation

When GMSCs reached 60% fusion, the medium was replaced with adipogenic induction culture medium consisting of MEM medium with 10 *μ*M human insulin, 1 *μ*M dexamethasone, 200 *μ*M indomethacin, and 0.5 mM 3-isobutyl-1-methylxanthine. The medium was replaced every three days for 14 days. Oil Red O staining was performed to detect intracellular lipid vacuole characteristic of adipocytes.

### 2.4. Culture and Proliferation Assay of the Schwann Cell

Three-day-old Sprague-Dawley (SD) mice were used. The bilateral sciatic nerves were cut, and its epicardium was removed. Sciatic nerve was collected and digested with 0.2% NB4 collagenase for 10 min at 37°C. Schwann cell culture medium (10% fetal bovine serum, 1% penicillin/streptomycin, and 10^−2^ M/mL forskolin) was added to resuspend the cells. Schwann cell was seeded in a cell culture flask. After 48 h, Schwann cell was digested and seeded in 96-well plates and 6-well plates cultured with exosomes (100 *μ*g/mL). After culture for 1, 3, and 5 days, 10 *μ*L of CCK solution was added to 96-well plates. The absorbance at 450 nm was measured with a microplate reader. Five days after culture, Schwann cell in 6-well plates was identified by immunofluorescence staining using rabbit anti-S100 antibody.

### 2.5. Isolation and Identification of Exosomes

GMSCs were cultured at 3 × 10^6^ cells/75 cm^2^ density with 15 mL medium containing exosomes-free fetal bovine serum for 48–72 h; then, the medium was collected in a 15 mL centrifugal tube and centrifuged at 3000×*g* for 10 min; the supernatant was then carefully collected and transferred to a new tube and placed on ice. The supernatant from 10 mL of cells was extracted, and 2.5 mL of the exosome extraction reagent (HieffTM Quick exosome isolation kit, 41201ES25) was added. The supernatant was mixed via vortex oscillation for 1 minute, and then placed at 4°C for 2 h. The tube containing the mixture was removed and centrifuged for 60 minutes at 4°C and 10000×*g*; then, the supernatant was discarded, and the pellet was collected. The centrifugal sediment was beaten uniformly with 100 *μ*L of PBS for mixing and transferred to a new 1.5 mL centrifugal tube. The 1.5 mL centrifugal tube containing exosome was centrifuged at 4°C for 2 min at 12000×g. The precipitation was discarded, and the supernatant was retained.

The protein content of the exosomes was detected by the BCA Protein Assay Kit (Thermo). The morphology of the exosomes was analyzed by transmission electron microscopy (JEM-1400 Plus). The size of exosomes was measured by nanoparticle tracking analyses (ZetaView). The exosomal characteristic markers, CD9 and CD63, were analyzed by Western blot analysis.

### 2.6. Isolation and Culture of Dorsal Root Ganglion (DRG) Cells with Exosomes

SD mice born within 24 hours were sacrificed. Then, 75% alcohol was used to sterilize the backs of mice; vertebrae were exposed and removed and then placed in DMEM-F12 medium containing 10% FBS. The vertebral body was cut centrally along the sagittal plane, and the DRG was extracted from the intervertebral foramen and placed in a new culture dish. The DRG epineurium was then peeled off and plated onto poly-lysine-coated glass coverslips in 6-well plates. The DRGs were cultured with exosomes (100 *μ*g/mL) in MEM medium (Gibco) supplemented with B-27 (Gibco) and GlutaMAX (Gibco). The control had no exosomes.

### 2.7. Immunofluorescence

After culture for 5 days, immunofluorescence staining was performed to evaluate the effect of exosomes on DRGs. First, DRG samples were fixed with paraformaldehyde for 30 minutes and then blocked with 10% goat serum for 1 hour. Next, mouse anti-neurofilament 200 (N0142; Sigma) and rabbit anti-S100 (S2644; Sigma) were applied as the primary antibodies for overnight incubation at 4°C. Goat anti-mouse IgG (Alexa Fluor® 488; Abcam) and goat anti-rabbit (Alexa Fluor® 594; Abcam) secondary antibodies were then incubated with the samples in the dark for 1 hour. Three randomly selected DRG samples from each group were used for statistical analysis. The three longest axons per quadrant were measured and used to calculate the mean length of each DRG axon using the Image-Pro Plus 6.0 image analysis software.

### 2.8. Animals and Surgical Procedures

Chitin conduits (diameter: 1.2 mm) were patented by the Peking University and China Spinning and Weaving Institute, and these can retain structure for at least 6 weeks in vivo and undergo complete biodegradation (please refer to Chinese patent ZL01136314 for technical details). Eight-week-old SD rats were provided by Beijing Weitong Lihua Co. Ltd. We followed the guideline for ethical review of animal welfare of China (GB/T 35892-2018), and all experiments complied with the relevant regulations of the Medical Ethics Committee of Peking University People's Hospital (approval no. 2102000024). Twenty-four SD rats weighing 200–220 g were randomly divided into three groups, with eight rats in each, as follows: the hollow chitin conduit group, chitin conduit plus GMSC-derived exosome group, and autograft group. All rats were anesthetized with a sodium pentobarbital solution (30 mg/kg body weight), and then, the right hind limb was prepared. The sciatic nerve was cut and exposed, and 10 *μ*L PBS was administered to the hollow chitin conduit group, retaining a 10 mm gap. The exosome group received 10 *μ*L PBS containing 10 *μ*g exosomes. Then, the muscle and skin were sutured. After waking, the rats were automatically fed food and drinking water and were housed with a 12-hour light/dark cycle and natural circulation.

### 2.9. Transmission Electron Microscopy (TEM) and Morphological Analyses

Twelve weeks after surgery, the regenerated nerve tissue samples were removed and fixed with 2.5% glutaraldehyde at 4°C for 2 hours. Samples were stained with 1% osmium acid, dehydrated with a gradient of acetone, embedded in Epon812 epoxy resin, and then cut into 700 nm thick semithin sections and 70 nm thick ultrathin sections. Uranyl acetate and lead citrate were used to stain the 70 nm thick ultrathin sections, which were observed by transmission electron microscopy (JEM-1400 Plus). Semithin sections were stained with 1% toluidine blue and observed by light microscopy. The number of regenerated axons was recorded based on five random images per animal. The diameter of myelinated axons and the thickness of the myelin sheath were measured using the Image-Pro Plus software. Five random axons were counted for each random sample of five random TEM images.

### 2.10. Muscle Weight and Muscle Fiber Remodeling

Twelve weeks after the operation, bilateral gastrocnemius muscles were removed from each group and fixed with muscle tissue fixative mainly containing formaldehyde, glacial acetic acid, and absolute ethanol (G1111; Servicebio) for 24 hours, immediately after weighing. Paraffin-embedded tissues were cut into 7 mL thick sections and subjected to Masson's trichrome staining. Five random fields were imaged for each sample by optical microscopy and quantitatively analyzed using Image-Pro Plus.

### 2.11. CatWalk Gait Analysis

The CatWalk XT 9.0 gait analysis system (Noldus, Wageningen, The Netherlands) was used to evaluate rat motor function recovery at 4, 8, and 12 weeks after surgery. The sciatic functional index (SFI) is a quantitative measure of sciatic nerve function. The formula is as follows: SFI = 109.5(ETS − NTS)/NTS − 38.3(EPL − NPL)/NPL + 13.3(EIT − NIT)/NIT − 8.8.

### 2.12. Electrophysiological Assessment

At 12 weeks after surgery, the regenerated sciatic nerves were exposed, while mice were under anesthesia. Parameters of the Medlec Synergy electrophysiological system (Oxford Instrument Inc., Abingdon, UK) were set at a stimulus intensity of 0.09 mA and a duration of 0.1 ms. The stimulating electrode was placed proximal to the sciatic nerve; the recording electrode was placed proximal and distal to the gastrocnemius muscle, and the reference electrode was placed on the gluteus maximus. Then, the CMAP latency and peak amplitude were calculated separately.

### 2.13. Statistical Analysis

All numerical data are expressed as the mean ± standard deviation. Statistical analysis was performed by calculating the analysis of variance (ANOVA) followed by Tukey's post hoc multiple comparison test. All data were analyzed using SPSS 17.0. Differences were considered statistically significant with a *p* value < 0.05.

## 3. Results

### 3.1. Identification of Human Gingiva-Derived Mesenchymal Stem Cells

**Figure 1:**
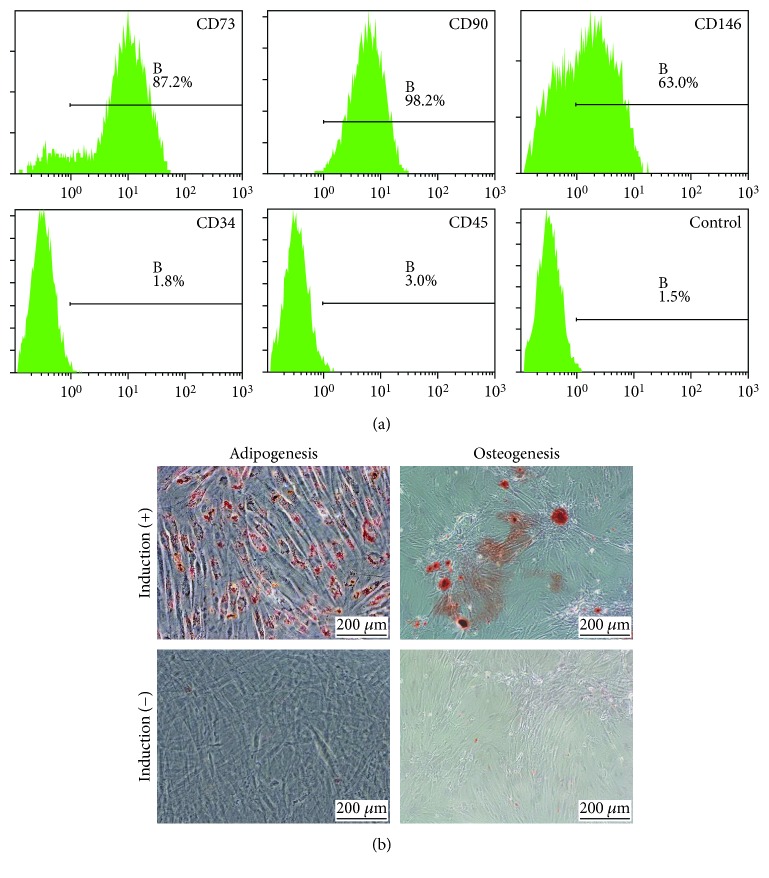
Characterization of GMSCs. (a) Flow cytometric analysis of surface markers of GMSCs. GMSCs were positive for MSC markers including CD73 (87.2%), CD90 (98.2%), and CD146 (63%) and were negative for hematopoietic stem cell markers, CD34 (1.8%) and CD45 (3.0%). (b) Representative images of adipogenic differentiation and osteogenic differentiation. The visual field was filled with red lipid droplets stained with Oil Red O solution in images of adipogenic differentiation. Calcium nodules with burrs were formed in images of osteogenic differentiation, which were stained red by Alizarin Red solution.

Flow cytometry analysis showed that GMSCs were positive for MSC markers including CD73 (87.2%), CD90 (98.2%), and CD146 (63%) and were negative for hematopoietic stem cell markers, CD34 (1.8%) and CD45 (3.0%). Also, CD106 is a marker of mesenchymal stem cells with strong immunoregulatory function (Figure 1(a)). Results of adipogenic and chondrogenic differentiation experiments demonstrated the differentiative potential of the GMSCs (Figure 1(b)).

### 3.2. Characterization of GMSC-Derived Exosomes

**Figure 2:**
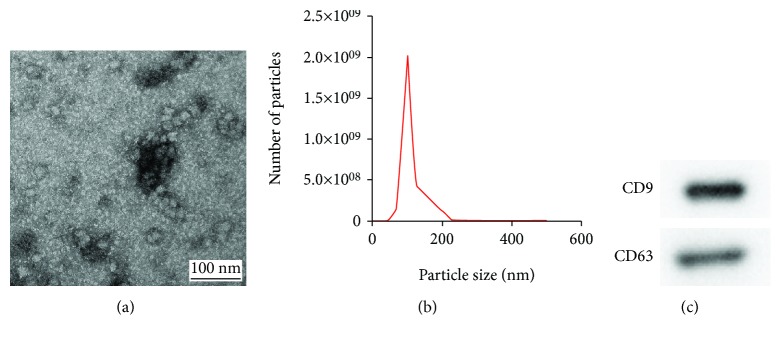
Characterization of exosomes from GMSC. (a) Representative images of exosomes from GMSC by TEM. (b) The size distribution profile of GMSC-derived exosomes. (c) Detection of exosomal markers (CD9 and CD63) by Western blot analysis.

The ultrastructure of objects was observed by TEM. As shown in Figure 2(a), the exosomes exhibited a circular structure by TEM. Further, the size distribution of the purified exosomes was measured with the nanoparticle tracking system, which showed that the peak diameter was 102 nm (Figure 2(b)). Finally, the results of Western blot analysis revealed that GMSC-derived exosomes expressed the exosome markers CD9 and CD63 (Figure 2(c)).

### 3.3. Effects of GMSC-Derived Exosomes on SCs and DRG

**Figure 3:**
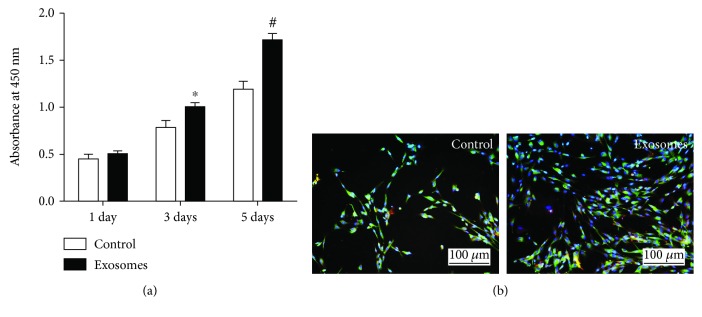
Exosomes promote Schwann cell proliferation. (a) Detection of the SC proliferation with CCK8. (b) S100 immunofluorescence of Schwann cells cultured with or without exosomes after 5 days. Results from 3 independent experiments with duplicates. Data are expressed as means ± SEM. Statistical significance was obtained with one-way ANOVA with Tukey's post hoc test. ^∗^*p* < 0.05, ^#^*p* < 0.01.

**Figure 4:**
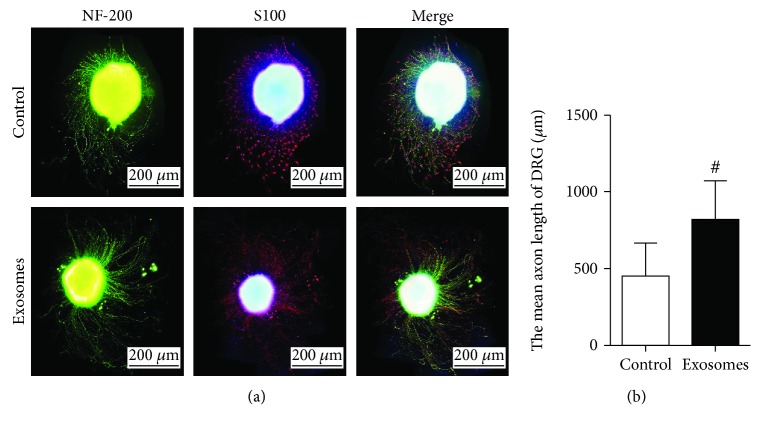
Exosomes enhance DRG neurite outgrowth. (a) DRGs were stained with NF200 (green) and S100 (red). (b) Statistical analysis of length of DRG neurite. Results from 4 independent experiments with duplicates. Data are expressed as means ± SEM. Statistical significance was obtained with one-way ANOVA with Tukey's post hoc test, ^#^*p* < 0.01.

Upon assessing the effect of GMSC-derived exosomes on SC outgrowth, CCK8 results showed no statistical difference between the two groups on the first day. On day 3, cell growth in the GMSC-derived exosome group was higher than that in the control group, and a statistical difference was noted between the groups (*p* < 0.05). On day 5, GMSC-derived exosomes were found to promote Schwann cell proliferation, and a statistical difference was noted between the two groups (*p* < 0.01) (Figure 3(a)). Immunofluorescence was also performed to show the Schwann cells of GMSC-derived exosomes and those of the control group on day 5 (Figure 3(b)). Also, immunofluorescence staining was performed to evaluate the effect of GMSC-derived exosomes on DRG neurite after culture for 5 days (Figure 4(a)). The length of DRG neurite cultured with GMSC-derived exosomes was significantly increased compared to the control (*p* < 0.01) (Figure 4(b)).

### 3.4. Histological Evaluation of Regenerative Nerves

**Figure 5:**
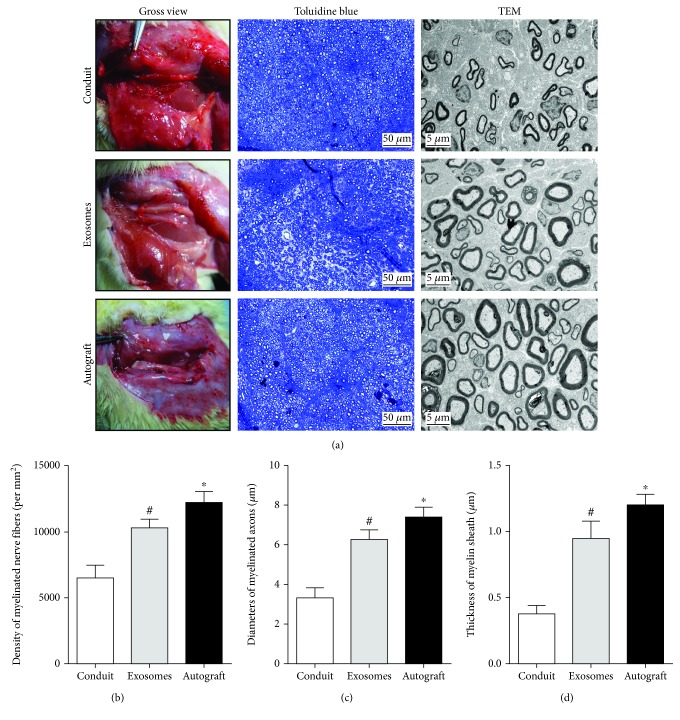
Evaluation of regenerated nerve fibers at 12 weeks after surgery. (a) Gross view, toluidine blue-stained transverse sections, and TEM images of the regenerated sciatic nerve. (b) Density of myelinated axons. (c) Diameters of myelinated axons. (d) Thickness of myelin sheath. Data are expressed as means ± SEM (*n* = 8). Statistical significance was obtained with one-way ANOVA with Tukey's post hoc test. ^∗^*p* < 0.05, ^#^*p* < 0.01.

Toluidine blue staining of regenerated tissue revealed the formation of myelinated nerve fibers, and statistical analysis was performed to compare the numbers of regenerated nerve fibers (Figure 5(a)). The mean diameter and myelin sheath thickness of myelinated nerve fibers were quantified after TEM. This confirmed an increased number of regenerated nerve fibers in the GMSC-derived exosome group compared to the control group (*p* < 0.05) (Figure 5(b)); further, nerve fiber diameter and myelin sheath thickness were also improved with exosome administration (*p* < 0.05) (Figures 5(c) and 5(d)). However, there was still a gap, compared to that observed in the autograft group.

### 3.5. Histological Evaluation of the Gastrocnemius

**Figure 6:**
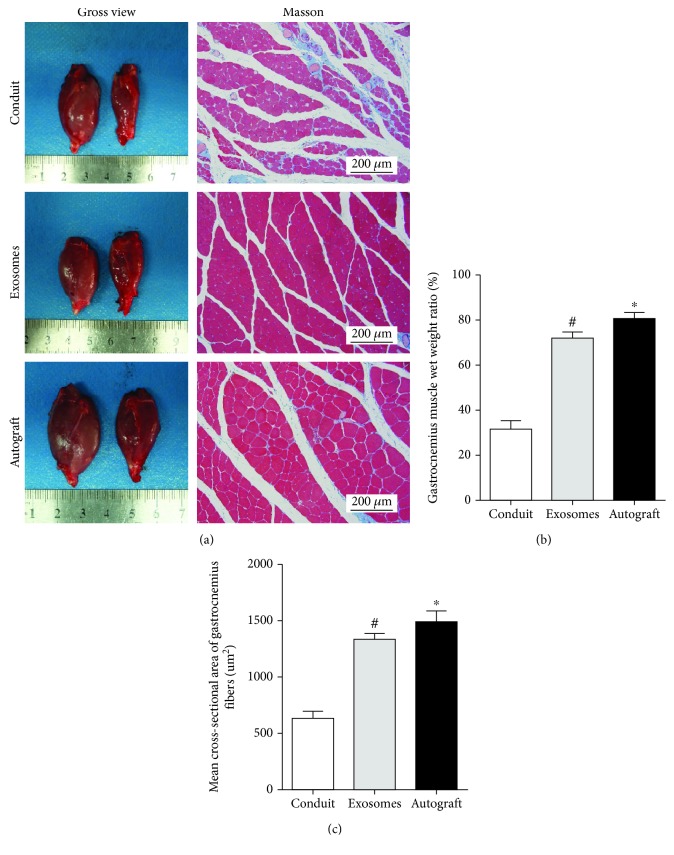
Reconstruction of muscle function at 12 weeks after surgery. (a) Gross images of gastrocnemius muscles and Masson's trichrome staining images. (b) Statistical analysis of the gastrocnemius muscle wet weight ratio. (c) Cross-sectional area of muscle fiber. Data are expressed as means ± SEM (*n* = 8). Statistical significance was obtained with one-way ANOVA with Tukey's post hoc test. ^∗^*p* < 0.05, ^#^*p* < 0.01.

The bilateral gastrocnemius muscles were isolated and assessed by Masson staining 12 weeks after the operation (Figure 6(a)). Cross-sectional images of muscles showed that cross-sectional muscle fibers in the GMSC-derived exosome group were better than those in the control group (*p* < 0.05) (Figure 6(b)), and the muscle wet weight ratio was also improved compared to that in the control group (*p* < 0.05) (Figure 6(c)), which was consistent with SFI results.

### 3.6. Electrophysiological Evaluation

**Figure 7:**
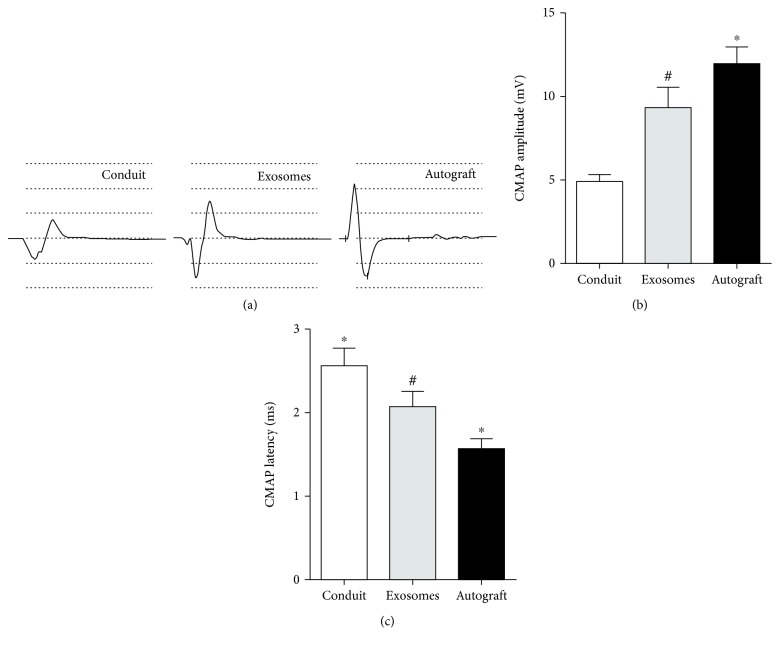
Electrophysiological detection of neuromuscular function at 12 weeks after surgery. (a) Representative CMAP recordings in each group. (b) Statistical analysis of CMAP amplitude and CMAP latency detected in each group. Data are expressed as means ± SEM (*n* = 8). Statistical significance was obtained with one-way ANOVA with Tukey's post hoc test. ^∗^*p* < 0.05, ^#^*p* < 0.01.

Electrophysiology was used to examine the electrical conduction of nerves (Figure 7(a)). Electrical conduction is related to the number of myelinated nerve fibers and the thickness of the myelin sheath. The latency of CMAP is related to the thickness of the myelin sheath, whereas the CMAP amplitude is consistent with the number of nerve fibers. The latency in the GMSC-derived exosome group was significantly shorter, and the amplitude was significantly higher than that in the control group (both *p* < 0.01) (Figure 7(b)).

### 3.7. Evaluation of Motor Function

**Figure 8:**
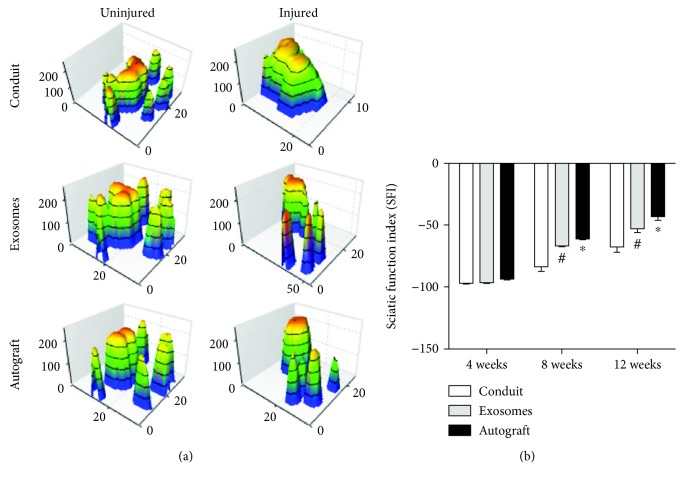
Motor functional recovery at 12 weeks by CatWalk gait analysis. (a) Representative 3D plantar pressure distribution of RH (injured) and LH (uninjured) of each group (by statistical analysis of SFI values). Data are expressed as means ± SEM (*n* = 8). Statistical significance was obtained with one-way ANOVA with Tukey's post hoc test. ^∗^*p* < 0.05, ^#^*p* < 0.01.

Motor function evaluation was performed using a mouse gait analysis system, which can record paw prints in real time.  Figure 8(a) showed a three-dimensional pressure diagram of paw prints. SFI was used to evaluate the recovery of motor function. At four-week postoperation, no statistical difference was found after comparing with the control group (*p* > 0.05) (Figure 8(b)). Eight weeks after surgery, the SFI in the GMSC-derived exosome group was improved compared to that in the control group and this difference was statistically significant (*p* < 0.05) (Figure 8(b)). Twelve weeks after surgery, the SFI in the GMSC-derived exosome group was similar to that in the autograft group and was significantly improved compared to that in the hollow conduit group (*p* < 0.05) (Figure 8(b)).

## 4. Discussion

Autologous nerve transplantation remains the gold standard for repairing peripheral nerve defects; however, it is necessary to uncover methods to replace this technique [32–34]. The local implantation of stem cells to treat peripheral nerve injury can promote axonal regeneration and myelin sheath formation. During the process of repair, stem cells secrete a variety of factors such as FGF, NGF, CNTF, BDNF, and GDNF, which can have a positive effect on neural cell survival and nerve regeneration [35–39]. Although stem cell-based therapies have shown beneficial effects on tissue regeneration, it has been reported that the main mechanism underlying stem cell-mediated tissue repair is paracrine rather than the differentiation of stem cells [40, 41]. Many studies have shown that the paracrine function of stem cells can be mediated by exosomes, which have significant potential to replace whole cell therapy as a new alternative method [15, 17, 19, 42].

Stem cell-derived exosomes deliver various molecules including cytokines, growth factors, signaling lipids, mRNA, and microRNAs. Moreover, exosomes, as intercellular mediators of communication, transfer proteins, lipids, and RNA to regulate various physiological and pathological processes [43]. In addition, the application of exosomes was shown to be safer than stem cell administration, which could overcome cellular immune rejection and carcinogenic mutations [44].

Gingival tissue is known to heal very quickly after injury, and scarring is rare, due to the functions of GMSCs. These cells have many advantages, including the fact that they are homogeneous, nontumorigenic, easily separated, and phenotypically stable [45]. In the present study, we successfully isolated GMSCs from human gingival tissue, and these cells showed osteogenic and adipogenic differentiation capabilities. Moreover, the isolated GMSCs expressed specific surface antigens including CD73, CD90, and CD146, but did not express hematopoietic antigens such as CD34 and CD45. These results were consistent with the previous reports [31].

GMSC transplantation for peripheral nerve regeneration after injury has been applied in several studies [25, 27, 28, 38]. Lai et al. and Court et al. suggested that exosomes might modulate neurite outgrowth in the CNS [46, 47]. However, to our knowledge, GMSC-derived exosomes have not been studied with respect to peripheral nerve defects. In our study, we also successfully isolated and characterized exosomes from GMSCs.

DRG culture in vitro is usually used to evaluate peripheral nerve regeneration. Our results showed for the first time that GMSC-derived exosomes can significantly promote DRG axonal growth. Lopez-Verrilli et al. found that exosomes derived from Schwann cells can significantly promote axonal growth in vitro and in vivo [48]. In the present study, we found that GMSC-derived exosomes could also significantly promote the proliferation of Schwann cells. Studies have reported that exosomes derived from adipose stem cells (ADMSCs) can be transferred into Schwann cells, which indicated that exosomes can be transferred between cells [49]. Also, exosomes of Schwann cell-like differentiated adipose stem cells express Gap43, Tau, Rac1, RhoA mRNAs, miR-18a, miR-182, miR-21, miR-222, and miR-1miRNAs associated with neural regeneration [50].

Using chitin conduits, we have performed different studies on small gap sutures in vivo [51–53]. Typically, the effect of hollow conduits on the repair of 10 mm nerve defects is poor, and therefore, we used chitin conduits combined with exosomes derived from GMSCs to repair 10 mm peripheral nerve defects in rats.

Twelve weeks after the operation, GMSC-derived exosomes not only enhanced the density and diameter of regenerated axons but also increased the thickness of the regenerated myelin sheath, which was consistent with the results of in vitro studies.

At the same time, electrophysiology results showed that the CMAP amplitude in the exosome group was significantly higher than that in the control group, whereas the CMAP latency was decreased in the exosome group compared to the control group. Gait analysis was also used to evaluate the restoration of neuromuscular function in rats. Our study showed that at 8 and 12 weeks after the operation, the SFI was improved in the GMSC-derived exosome group compared to the control group. This was consistent with histological and electrophysiological data. However, regenerated nerve grows into the muscle until 4 weeks, and there was no statistical difference between the groups at this time.

In this study, 12 weeks after surgery, muscle histological data showed that the muscle wet weight and muscle fiber cross-sectional area were increased in the exosome group compared to the control group, indicating improved restoration of neuromuscular function, indirectly verifying the results of gait analysis.

Although GMSC-derived exosomes in chitin conduits have achieved good results in the treatment of peripheral nerve injury, the extraction method of exosomes needed further optimization to get more exosomes. Also, one remaining question was how gingival stem cell-derived exosomes function. Kou et al. found [54] that GMSC-derived exosomes have more protein content, including cytokines that can dampen the function of IL-1RA, which has an anti-inflammatory effect. Coincidentally, IL-1RA is a known biological agent that can be used for the treatment of rheumatoid arthritis. Upon injection of IL-1RA into the wound, the wound healing rate was found to be greatly accelerated. In that study, the researchers found that GMSCs from diabetic mice secreted fewer exosomes than those from normal mice, resulting in a decrease in IL-1RA production. Moreover, the healing rate of diabetic mice was improved by applying IL-1RA from the GMSCs of healthy mice. Therefore, IL-1RA might play a crucial role in the repair of peripheral nerve injury. Our next study will examine whether IL-1RA promotes axonal growth and reduces scar formation.

## 5. Conclusions

In summary, the effect of exosomes on intercellular interactions is an exciting research area. Our results suggest that GMSC-derived exosomes combined with biodegradable chitin conduits represent a promising new approach for nerve regeneration.

## Data Availability

All of the data and materials are available.
